# Hemodynamic effects of high frequency oscillatory ventilation versus conventional mechanical ventilation in term neonates with persistent pulmonary hypertension: a single-center randomized trial

**DOI:** 10.1186/s13052-026-02272-z

**Published:** 2026-05-11

**Authors:** Doaa El Amrousy, Hassan Koura, Noura Abdou, Mostafa Awny, Abdelrahman Elmashad

**Affiliations:** Pediatric Department, Faculty of Medicine, Tanta University, Tanta, Egypt

**Keywords:** HFOV, CMV, Neonates, Hemodynamics

## Abstract

**Background:**

We aimed to evaluate the hemodynamics in full-term neonates with persistent pulmonary hypertension (PPH) on high frequency oscillatory ventilation (HFOV) versus conventional mechanical ventilation (CMV).

**Methods:**

This trial was conducted on 64 full-term neonates with PPH who needed MV and were randomly divided into two groups: group I: 32 neonates with PPH on HFOV, and group II: 32 neonates with PPH on CMV. Two neonates weaned early from group I, and another two neonates died in group II; they were excluded from the study. Heart rate (HR), systolic, diastolic, and mean blood pressure (SBP, DBP, MBP) were recorded. Echocardiography was performed to measure right ventricular diameter (RVD), right atrial diameter (RAD), mean pulmonary artery diameter (MPA), left ventricular dimensions, LV systolic function, tricuspid pressure gradient (TR PG), systolic pulmonary artery pressure (SPAP), tricuspid annulus plane systolic excursion (TAPSE), superior vena cava (SVC) flow, right and left ventricular output (RVO, LVO). Cerebral ultrasound was done to measure the resistive index (RI) of middle cerebral artery (MCA) and to detect intraventricular hemorrhage (IVH). All measurements were performed at day 1, 3, and 7.

**Results:**

HR, RVD, RAD, and MPA significantly decreased, but SBP, DBP, MBP, LVO, LV dimensions, and function significantly increased in both groups at day 7. SPAP and TR PG decreased significantly at day 3 and 7; however, TAPSE and RVO increased significantly at day 3 and 7 in both groups. LVO and RVO increased more significantly at day 7 in the CMV group. SVC flow didn’t differ significantly in both groups on follow-up. RI of MCA significantly decreased in the HFOV group only at day 7. Moreover, IVH was comparable in both groups.

**Conclusion:**

HFOV and CMV have positive comparable effects on hemodynamics in full-term neonates with PPH.

**Clinical trial registration:**

The study was registered at pan African clinical trial registry with registration number (PACTR202104624409011) with registration date 6th April 2021. Link for trial registration (https://pactr.samrc.ac.za/TrialDisplay.aspx?TrialID=15789).

## Introduction

Persistent Pulmonary Hypertension of Newborn (PPHN) is a condition characterized by marked pulmonary hypertension resulting from elevated pulmonary vascular resistance (PVR) and altered pulmonary vasoreactivity [1].

However, PPHN is a less common cause of respiratory distress in newborns, but it has a significant impact on morbidity and mortality [2]. PPHN is associated with meconium aspiration syndrome, pneumonia, sepsis, pulmonary hypoplasia, congenital diaphragmatic hernia, and maladaptation of the pulmonary vascular bed both in utero and ex utero [3].

High-frequency oscillatory ventilation (HFOV) has been used with increasing frequency for PPHN. Moreover, HFOV improves oxygenation at lower mean airway pressures and ventilation of infants with PPHN, as compared to conventional mechanical ventilation (CMV) [4, 5].

Although HFOV is used frequently in neonatal intensive care units (NICU), there are few studies about its hemodynamic effects in different diseases [6, 7]. The results of studies using different experimental models have not been consistent, so it remains unclear as to what, if any, hemodynamic consequences should be anticipated when switching to HFOV. A better understanding of the hemodynamic effects of HFOV would facilitate clinical decision making.

Hemodynamic assessment of neonates with PPH on HFOV has not studied before. Thus, this study aimed to evaluate the hemodynamics in full-term neonates with PPH on HFOV versus CMV.

## Methods

This clinical trial was conducted at NICU, Pediatric Department, Tanta University Hospitals. The study was approved by the local ethical committee of the Faculty of Medicine, Tanta University with approval code (34297/11/20). Written parental consent was signed before the enrollment of the studied neonates. The study was conducted in accordance with the Helsinki declaration. This clinical trial was registered at Pan African Clinical Trial Registry with registration number PACTR202104624409011.

Inclusion criteria: full-term neonates with a confirmed echocardiographic diagnosis of PPHN requiring mechanical ventilation.

Exclusion criteria: Neonates with major congenital abnormalities, congenital heart diseases, neonatal sepsis, arrhythmias, maternal diabetes, neonates with hydrops fetalis, neonates with severe intraventricular hemorrhage, preterm neonates, and neonates with perinatal asphyxia.

Sixty-four full term neonates with PPH who needed mechanical ventilation were included in the study during the period from April 2021 to December 2022. The enrolled neonates were randomly divided into two groups:

- Group 1: included 32 full-term neonates with PPH on HFOV.
- Group 2: included 32 full-term neonates with PPH on CMV.

Randomization of the patients was carried out by an independent statistician who used simple randomization through a randomization table created by a computer software program, and sealed opaque envelopes with sequential numbers were used for allocation concealment. Randomization was made after birth immediately after echocardiographic confirmation of PPHN. After obtaining written consents, the sealed opaque envelope was opened, and the patient was included in the respective group. The outcome assessors were blinded to the study groups.

All neonates were subjected to full history taking, thorough clinical examination with special emphasis on the weight, length, physical signs, Apgar score at 1 and 5 min, and New Ballard score for estimation of gestational age. Heart rate (HR), systolic blood pressure (SBP), and diastolic blood pressure (DBP), mean blood pressure (MBP), Downes’ scoring to assess the degree of respiratory distress were also recorded. Routine laboratory investigations included complete blood picture, C-reactive protein, liver and renal function tests, random blood sugar, and arterial blood gases were performed.

### Ventilatory settings

HFOV: (Fabian, Acutronic medical system and SLE 5000, UK) Mean airway pressure (MAP) was optimized for each neonate according to the baseline observations using an open lung approach, aiming to decrease FIO_2_ to below 0.4 as soon as possible. We set the oscillatory frequency between 10 and 15 Hz according to the patient’s weight. We adjusted the inspiration/expiration ratio to 1:2. Moreover, we initially adjusted the amplitude as double the mean level then we changed it according to the level of PCO_2_ and the tidal volume was the result of the adjustment of amplitude. We adjusted oxygenation by increasing MAP and FIO_2_. We knew that optimum lung volume is reached when an anteroposterior chest X-ray reveals an inflation close to the eighth posterior rib.

CMV: was provided by ventilators with positive pressure ventilation or trigger modes such as pressure-controlled ventilation (PCV), synchronized intermittent mandatory ventilation (SIMV), and SIMV with pressure support (SIMV + PS). We adjusted peak inspiratory pressure (PIP) between 20 and 25 cm H_2_O, FIO_2_ between 60 and 80%, positive end-expiratory pressure (PEEP) = 5 cm H_2_O, and ventilator rate between 40 and 60 rpm to achieve a tidal volume of 4–6 ml/kg. These initial parameters were changed according to the results of blood gases and the condition of every neonate.

Ventilator weaning criteria: Neonates were considered for weaning from mechanical ventilation when they maintained adequate oxygenation and ventilation with reduced ventilatory support, including lower mean airway pressure (less than 8–10 cm H_2_O) in HFOV or PIP (less than 15–20 cm H20) and PEEP (less than 5 cm H2O) in CMV, stable hemodynamics, and minimal respiratory distress. The decision to wean was guided by arterial blood gases, clinical examination, oxygenation index (OI) to be less than 10, PaO_2_/FIO_2_ ratio (more than 150–200), FIO_2_<0.4, stable systemic blood pressure, and improving SPAP.

Oxygenation Assessment: Oxygenation and respiratory response were monitored through arterial blood gases (PaO₂, PaCO₂), FiO₂, oxygenation index (OI) and PaO₂/FiO₂ ratio.

Rescue strategy in non-responders: For neonates who did not achieve adequate oxygenation or showed worsening clinical status, rescue strategies included escalation of ventilatory support, such as increasing MAP or amplitude (HFOV), increase PEEP/PIP (CMV), optimization of the dose of sildenafil, or inotrope adjustment.

### Echocardiography

The performed echocardiographic study included a full morphologic and hemodynamic assessment of cardiac anatomy and physiology using a segmental approach. Echocardiography examinations were performed on days 1, 3, and 7 of ventilation. Examination was performed for all studied neonates using Siemens Acuson X300 ultrasound machine (Siemens Health Care GmbH) with a 7 MHz transducer. Echocardiographic examination was done for all included neonates according to the American Society of Echocardiography (ASE) guidelines [8]. Left ventricular end-diastolic dimension (LVEDD) and LV end-systolic dimension (LVESD) were measured. LV fraction shortening (LV FS) was assessed as follows (LVFS%) = (LVEDD − LVESD) / LVEDD × 100. LV ejection fraction (LV EF) was also assessed as follows LVEF= (LVEDD)^3^ - (LVESD)^3^ / (LVEDD)^3^ × 100.

All cardiac hemodynamic measurements were also performed, such as superior vena cava (SVC) flow, left ventricular output (LVO), right ventricular output (RVO), and systolic pulmonary artery pressure (SPAP).

SVC flow was measured using the method described by Kluckow and Evans [9] through the sagittal subcostal view. Assessment of LVO involves measuring the mean velocity of blood flow across the ascending aorta from an apical five-chamber view using pulsed Doppler and determining the diameter of the aortic root from a parasternal long-axis view using M‐mode method. The area under the waveform of the aortic systolic beat can be used to calculate the velocity time integral (VTI). LVO = (π × aortic diameter^2^/4 × VTI × heart rate) / weight and is expressed in ml/kg/min. RVO was assessed using a similar approach prescribed in LVO with the pulmonary diameter measured at the hinge‐points of the pulmonary valve during cardiac systole from either the tilted parasternal long axis or the parasternal short axis view. SPAP was assessed in the absence of RVO obstruction bases on the velocity of the tricuspid regurgitate jet using the simplified Bernoulli equation, as SPAP = 4 (tricuspid regurgitate peak velocity)^2^ + right atrial (RA) pressure. If tricuspid regurgitation was not present, we used pulmonary artery acceleration time (PAAT) to estimate SPAP. PAAT was obtained by placing the Doppler cursor in the center of the distal RVOT just below the pulmonary valve annulus in the parasternal short axis view. SPAP = 82.6 − 0.58 × PAAT + RA pressure. Tricuspid annular plane systolic excursion (TAPSE) was determined by the difference in the distance from tricuspid lateral annulus to transducer interface along a parallel line in diastole and systole from 2D apical four-chamber views [10].

An expert pediatric cardiologist who was blinded to the study groups performed the echocardiographic examinations. As per the protocol of our unit, all included neonates with PPHN received sildenafil, initiated after echocardiographic confirmation of PPHN and optimization of mechanical ventilation, in a dose of 0.5-2 mg/kg/dose up to 4 times per day.

### Cerebral ultrasound examinations

Transcranial ultrasound study was performed using Siemens Acuson 300 ultrasound machine (Siemens Health Care GmbH, Erlangen, Germany) with a 7 MHz Transducer to detect complications of MV as IVH. Intraventricular hemorrhage was graded according to Papile classification into four grades [11].

The temporal bone approach is best for the middle cerebral artery (MCA) because it is parallel to flow. The resistive index (RI) of MCA ranges from 0.6 to 0.8 [12]. The cranial ultrasound was performed at day 1, 3, and 7 of starting ventilation.

RI measurements are typically acquired in the sagittal plane through the anterior fontanelle, utilizing curved and linear array transducers with frequencies ranging between 6.5 and 9 MHz. For RI data collection, one of the anterior cerebral arteries was sampled using a Doppler technique with angle correction. RI was calculated automatically using the following formula: (peak systolic velocity -diastolic velocity) /peak systolic velocity [13].

The primary outcome was to assess RVO changes in full-term neonates with PPHN after the use of MV between the two groups at different follow-up points (days 1, 3, and 7). The secondary outcomes were to assess cardiac function, dimensions, SPAP, LVO, SVC flow, and the incidence of IVH after HFOV use in these neonates.

### Statistical analysis

The sample size calculation was performed using G power program. We needed 60 full-term infants, 30 in each group, to achieve a power 95% with an effect size 0.98 and an alpha error 0.05. Data were analyzed using the statistical package for the social science (SPSS) V.21 program. Continuous data were presented as mean and standard deviation (SD), while categorical data were presented in the form of numbers and percentages. The Shapiro-Wilk test was used to assess the normality of the data. For categorical data, comparison was done using χ2 test. For normally distributed quantitative data, Student t-test was used to compare means between the two studied groups. Repeated measures were compared using one‐way analysis of variance (ANOVA). A post hoc test was used to detect significance between serial measurements. A P-value of < 0.05 was considered statistically significant.

## Results

**Fig. 1:**
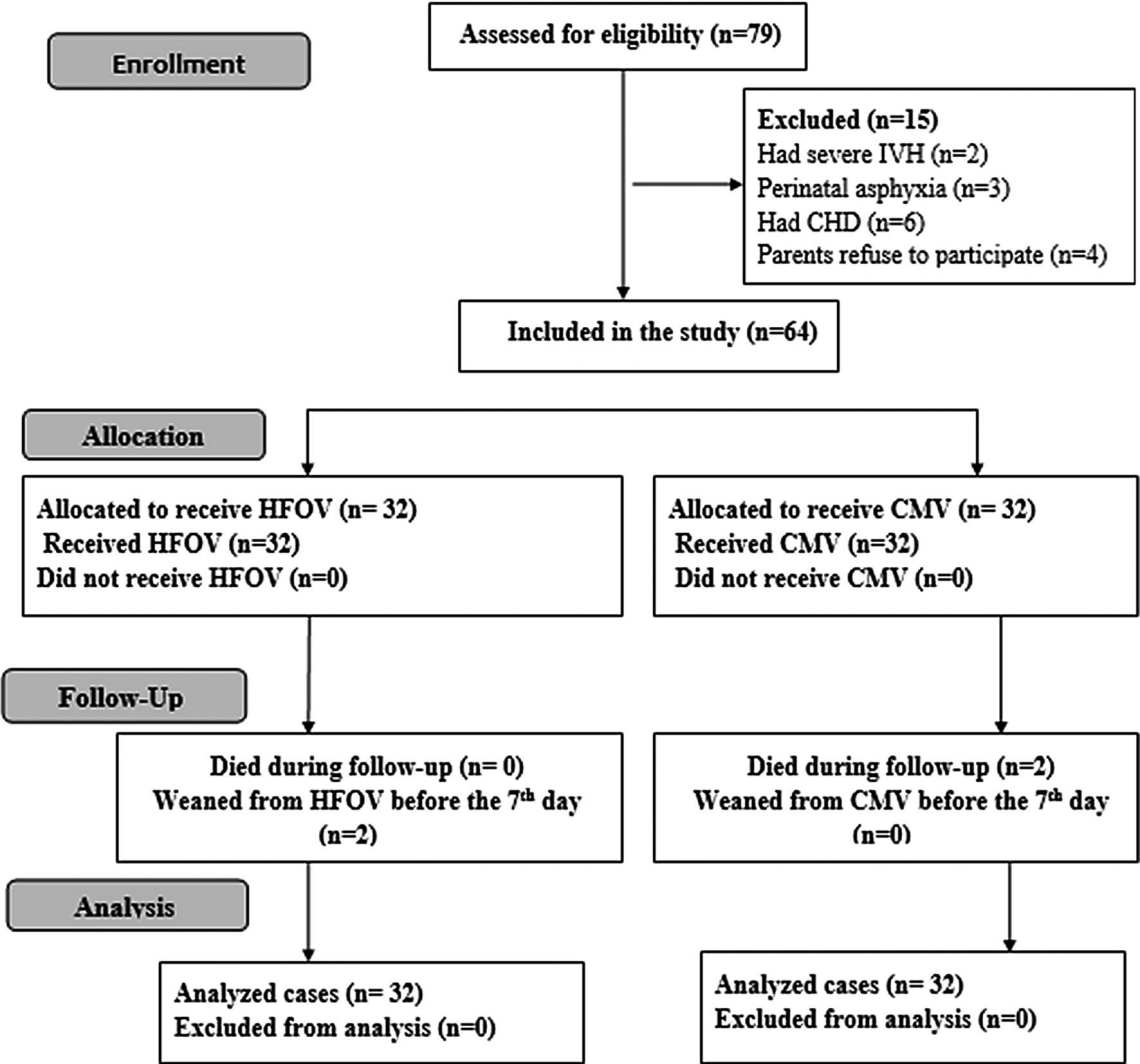
The flow chart of the study

The flow chart of the study is shown in Fig. 1. We screened 79 neonates with PPHN for eligibility; 15 were excluded, and 64 were enrolled and randomly assigned into two groups, each with 32 patients. Two patients in the HFOV group were weaned before 7th day, and another two patients died during follow-up in the CMV group. Intention-to-treat analysis was performed.

The characteristics of the studied groups are shown in Table 1. Both groups are comparable as regards gestational age, sex, mode of delivery, weight, length, Downes’ score, APGAR score 1 & 5 min, New Ballard score, blood gas, and the need for inotropes.

**Table 1:** Characteristics, measurements, and blood gas of the studied groups

	HFOV group (*n* = 32)		CMV group (*n* = 32)		*P*
Sex					
Male Female	16 16	(50%) (50%)	17 15	(53.1%) (46.9%)	0.537
Gestational age (weeks)	37.7 ± 0.9		37.6 ± 0.7		0. 910
Mode of delivery					
NVD CS	13 19	(40.6%) (59.4%)	16 16	(50%) (50%)	0.140
APGAR 1 min	4.8 ± 0.8		4.7 ± 0.6		0.712
APGAR 5 min	7.9 ± 0.8		7.8 ± 0.9		0.645
weight (kg)	3.2 ± 0.4		3.4 ± 0.4		0. 180
length (cm)	50.3 ± 0.4		49.8 ± 0.6		0. 726
Downes ' score	7.5 ± 0.5		7.7 ± 0.5		0. 208
NewBallard score	38.0 ± 0.9		37.7 ± 0.9		0. 673
Need for inotropes: N (%)	12 (40%)		10 (33.3%)		0.863
pH	7.29 ± 0.04		7.29 ± 0.06		0.951
PCO_2_	54.14 ± 4.06		50.07 ± 9.10		0.184
HCO_3_	14.74 ± 3.28		14.12 ± 2.19		0.830

HFOV: high-frequency oscillatory ventilation, CMV: conventional mechanical ventilation, NVD: normal vaginal delivery, CS: cesarean section

Table 2 showed that HR significantly decreased in both groups at days 3 and 7 compared to their values at day 1, with no significant difference between the two groups. SBP, DBP, and MBP significantly increased in both groups at day 7 compared to their values at day 1 and day 3, with no significant difference between the two groups. Moreover, PH and HCO_3_ significantly increased at day 3 and day 7 after ventilation compared to their values at day 1 in both groups, with no significant difference between the two studied groups. However, PCO_2_ significantly decreased at day 3 and day 7 after ventilation compared to its value at day 1 in both groups, with no significant difference between the two studied groups.

**Table 2:** Comparison between the two studied groups according to vital sings and blood gas

Vital signs	Time points	HFOV group	CMV group	Cohen’s d	95% CI	*P*
HR (b/m)	**Day 1**	144.3 ± 7.4	140.4 ± 10.7	0.44	-0.08-0.95	0.102
	**Day 3**	130.5 ± 10.1*	127.4 ± 8.1*	0.34	-0.17-0.85	0.198
	**Day 7**	117.6 ± 3.6*†	115.6 ± 5.7*†	0.42	-0.09-0.93	0.128
SBP (mmHg)	**Day 1**	63.3 ± 5.3	66.3 ± 5.3	-0.57	-1.08-0.05	0.451
	**Day 3**	65.9 ± 6.0	69.9 ± 5.6	-0.69	-1.21-0.17	0.09
	**Day 7**	80.4 ± 3.4*†	79.9 ± 3.6*†	0.14	-0.37-0.65	0.550
DB (mmHg)	**Day 1**	38.8 ± 4.4	39.1 ± 7.7	-0.05	-0.56-0.46	0.321
	**Day 3**	40.3 ± 5.0	42.3 ± 4.8	-0.41	-0.92-0.10	0.432
	**Day 7**	45.3 ± 3.3*†	47.0 ± 4.0*†	0.46	-0.97-0.05	0.090
MBP(mmHg)	**Day 1**	47.5 ± 4.6	48.2 ± 6.2	0.13	-0.64-0.38	0.512
	**Day 3**	48.7 ± 5.5	51.4 ± 5.3	-0.50	-1.01-0.01	0.138
	**Day 7**	56.7 ± 3.2*†	58.2 ± 4.1*†	-0.41	-0.92-0.10	0.081
Ph	**Day 1**	7.29 ± 0.04	7.29 ± 0.06	0.00	-0.51-0.51	0.951
	**Day 3**	7.35 ± 0.04*	7.37 ± 0.04*	-0.50	-1.01-0.01	0.846
	**Day 7**	7.38 ± 0.04*†	7.39 ± 0.04*†	-0.25	-0.76-0.26	0.911
PCO_2_	**Day 1**	54.14 ± 4.06	52.07 ± 2.10	0.64	-0.12-1.16	0.184
	**Day 3**	49.69 ± 2.93*	48.21 ± 1.76*	0.61	-0.09-1.13	0.090
	**Day 7**	48.52 ± 2.34*	47.83 ± 1.45*	0.36	-0.15-0.87	0.138
HCO_3_	**Day 1**	14.74 ± 3.28	14.12 ± 2.19	0.33	-0.51-2.37	0.830
	**Day 3**	17.72 ± 2.68*	19.41 ± 3.38*	0.55	-3.27-0.11	0.572
	**Day 7**	19.62 ± 1.72*	20.28 ± 1.65*	0.39	-1.53-0.21	0.340

P: denoted difference between HFOV and CMV groups, * denotes significant difference compared to day 1, † denotes significant difference compared to day 3, CI: confidence interval, HFOV: high-frequency oscillatory ventilation, CMV: conventional mechanical ventilation, HR: heart rate, SBP: systolic blood pressure, DBP: diastolic blood pressure, MBP: mean blood pressure

Table 3 showed that RVD, RAD, and MPA significantly decreased in both groups at day 7 compared to their values at day 1 and day 3, with no significant difference between the two groups. Furthermore, TR PG and SPAP started to decrease significantly in both groups at day 3 and day 7 compared to their values at day 1, with no significant difference between the two groups. However, SVC increased in both groups but did not reach a significant level, and its values were comparable in both groups at days 1,3, and 7. On the other hand, TAPSE started to increase significantly in both groups at day 3 and day 7 compared to its values at day 1, with no significant difference between the two groups. Moreover, RVO started to increase significantly in both groups at day 3 and day 7 compared to their values at day 1, with more significant values in the CMV group compared to the HFOV group.

**Table 3:** Comparison between the two studied groups according to echocardiographic parameters of the right ventricle

Parameters	Time points	HFOV group	CMV group	Cohen’s d	95% CI	*P*
RVD (mm)	**Day 1**	18.9 ± 1.9	18.0 ± 2.5	0.41	-0.25-2.05	0.151
	**Day 3**	17.1 ± 1.6	16.3 ± 1.9	0.46	-0.11-1.71	0.617
	**Day 7**	14.4 ± 1.4*†	13.9 ± 1.2*†	0.38	-0.17-1.17	0.171
RAD (mm)	**Day 1**	20.5 ± 1.8	19.7 ± 2.6	0.31	-0.46-1.86	0.149
	**Day 3**	18.9 ± 1.7	17.1 ± 2.3	0.49	-0.75-2.85	0.08
	**Day 7**	16.2 ± 1.3*†	15.7 ± 1.5*†	0.36	-0.23-1.23	0.125
MPA (mm)	**Day 1**	10.9 ± 2.1	10.8 ± 1.9	0.05	-0.96-1.16	0.360
	**Day 3**	9.9 ± 1.7	10.0 ± 1.2	-0.07	-0.86-0.66	0.140
	**Day 7**	8.0 ± 0.8*†	8.3 ± 0.9*†	-0.35	-0.74-0.14	0.240
TRPG(mmHg)	**Day 1**	61.5 ± 8.2	57.9 ± 7.6	0.44	-0.59-7.59	0.101
	**Day 3**	48.3 ± 7.6*	45.3 ± 7.3*	0.40	-0.85-6.85	0.140
	**Day 7**	21.9 ± 2.4*†	22.9 ± 2.5*†	-0.41	-2.27-0.27	0.119
SPAP(mmHg)	**Day 1**	66.4 ± 8.2	62.7 ± 7.5	0.46	-0.46-7.66	0.080
	**Day 3**	53.3 ± 7.6*	49.3 ± 8.0*	0.51	-0.03-8.03	0.058
	**Day 7**	26.9 ± 2.5*†	27.9 ± 2.5*†	-0.40	-2.29-0.29	0.119
TAPSE	**Day 1**	5.7 ± 1.1	6.2 ± 1.6	-0.36	-1.21-0.21	0.142
	**Day 3**	7.2 ± 1.0*	7.5 ± 1.1*	-0.29	-0.84-0.24	0.253
	**Day 7**	8.6 ± 1.4*†	8.1 ± 0.7*†	0.45	-0.07-1.07	0.072
SVC flow (ml/kg/min)	**Day 1**	149 ± 35	148 ± 30	0.06	-14.85-18.85	0.586
	**Day 3**	150 ± 39	155 ± 37	-0.13	-24.65-14.65	0.829
	**Day 7**	155 ± 30	160 ± 42	-0.14	-23.86-13.86	0.180
RVO (ml/kg/min)	**Day 1**	310 ± 38	345 ± 68	0.44	-63.47- 6.53	0.514
	**Day 3**	351 ± 35*	422 ± 33*	-2.09	-88.58- -53.42	0.009
	**Day 7**	472 ± 41*†	555 ± 27*†	-2.39	-100.94- -65.06	0.002

P: denoted difference between HFOV and CMV groups, * denotes significant difference compared to day 1, † denotes significant difference compared to day 3, HFOV: high-frequency oscillatory ventilation, CMV: conventional mechanical ventilation, CI: confidence interval, RVD: right ventricular diameter, RAD: right atrial, TR PG: tricuspid pressure gradient, SPAP: systolic pulmonary artery pressure, TAPSE: tricuspid annulus plane systolic excursion, SVC: superior vena cava, RVO: right ventricular output

Table 4 showed that LVEDD, LVESD, LV FS, and LV EF significantly increased in both groups at day 7 compared to their values at day 1 and day 3, with no significant difference between the two groups. Interestingly, LVO significantly increased in both groups at day 7 compared to their values at day 1 and day 3, which was more marked in CMV group compared to HFOV group.

**Table 4:** Comparison between the two studied groups according to echocardiographic parameters of the left ventricle

Parameters	Time points	HFOV group	CMV group	Cohen’s d	95% CI	*P*
LVEDD (mm)	**Day 1**	18.5 ± 0.8	18.7 ± 1.2	-0.20	-0.73-0.33	0.288
	**Day 3**	20.7 ± 1.1	20.7 ± 1.0	0.00	-0.54-0.54	0.900
	**Day 7**	21.5 ± 1.0*†	21.2 ± 1.0*†	0.30	-0.22-0.82	0.210
LVESD (mm)	**Day 1**	11.6 ± 0.7	11.8 ± 0.9	-0.25	-0.62-0.22	0.307
	**Day 3**	14.2 ± 1.1	13.9 ± 1.0	0.29	-0.24-0.84	0.277
	**Day 7**	15.1 ± 1.0*†	14.7 ± 1.0*†	0.40	-0.12-0.92	0.177
LVFS (%)	**Day 1**	28.9 ± 2.2	29.1 ± 2.7	-0.08	-1.47-1.07	0.214
	**Day 3**	31.6 ± 2.7	33.0 ± 2.2	-0.57	-2.67- -0.13	0.037
	**Day 7**	36.5 ± 1.2*†	36.7 ± 1.6*†	-0.14	-0.93-0.53	0.521
LV EF (%)	**Day 1**	63.8 ± 2.8	63.4 ± 4.1	0.11	-1.41-2.21	0.628
	**Day 3**	65.6 ± 3.0	65.1 ± 3.1	0.16	-1.08-2.08	0.605
	**Day 7**	68.8 ± 2.6*†	68.2 ± 1.9*†	0.26	-0.58-1.78	0.299
LVO (ml/kg/min)	**Day 1**	399 ± 36	422 ± 67	-0.43	-50.8-4.8-	0.683
	**Day 3**	410 ± 37	474 ± 66	-0.2	-91.65- 36.35	0.065
	**Day 7**	494 ± 45*†	541 ± 38*†	-1.13	-68.53- -25.47	0.001

P: denoted difference between HFOV and CMV groups, * denotes significant difference compared to day 1, † denotes significant difference compared to day 3, HFOV: high-frequency oscillatory ventilation, CMV: conventional mechanical ventilation, CI: confidence interval, LVESD: left ventricular end-systolic diameter, LVEDD: left ventricular end-diastolic diameter, LV FS: left ventricular fraction shortening, LV EF: left ventricular ejection fraction, LVO: left ventricular output

RI of MCA decreased significantly in the HFOV group at day 7 compared to its values at day 1 and day 3; however, RI of MCA didn’t differ in the CMV group on follow-up. Furthermore, RI of MCA was significantly lower in the HFOV group compared to the CMV group at day 7. Interestingly, both groups were comparable regarding the incidence of IVH in the studied neonates at days 1, 3, and 7 as shown in Table 5.

**Table 5:** Comparison between the two studied groups according to cranial ultrasound

Parameters	Time points	HFOV group	CMV group	*P*
RI of MCA	**Day 1**	0.61 ± 0.03	0.63 ± 0.03	0.531
	**Day 3**	0.61 ± 0.03	0.60 ± 0.04	0.426
	**Day 7**	0.57 ± 0.03*†	0.60 ± 0.02	˂0.001
Grades of IVH	**Day 1**	*N* = 32	*N* = 32	
	No IVH	26 (81.3%)	27 (84.4%)	0.362
	1	6 (18.7%)	5 (15.6%)	
	2	0 (0%)	0 (0%)	
	**Day 3**	*N* = 32	*N* = 32	
	No IVH	25 (78.1%)	27 (84.4%)	0.310
	1	6 (18.8%)	5 (15.6%)	
	2	1 (3.1%)	0 (10.0%)	
	**Day 7**	*N* = 32	*N* = 30 (2 died)	
	No IVH	25 (78.1%)	25 (83.3%)	0.154
	1	5 (15.6%)	4 (13.3%)	
	2	2 (6.3%)	1 (3.3%)	

P: denoted difference between HFOV and CMV groups, * denotes significant difference compared to day 1, † denotes significant difference compared to day 3, NS: non-significant, HFOV: high-frequency oscillatory ventilation, CMV: conventional mechanical ventilation, RI MCA: resistive index of middle cerebral artery, IVH: intraventricular hemorrhage

The duration of ventilation and NICU stay was comparable in both groups. One neonate died in the HFOV group, while four neonates died in the CMV group, with no significant difference (Table 6).

**Table 6:** Clinical outcomes in the studied groups

Parameters	HFOV group	CMV group	*P* value
Duration of ventilation (days)	7.8. ± 0.9	8.0 ± 1.1	0.129
Duration of ICU stay (days)	15.0 ± 2.1	15.8 ± 2.3	0.401
Survival, n (%)	31 (96.9%)	28 (87.5%)	0.36

NICU: neonatal intensive care unit

## Discussion

PPHN represents a complex cardiopulmonary disorder characterized by elevated PVR, right to left shunt, and compromised systemic oxygen delivery [1, 2]. Our study is the first to assess the hemodynamic effects of HFOV versus CMV on full-term neonates with PPHN in the context of standardized sildenafil therapy. We found that both ventilation strategies significantly decreased PVR, reduced right-sided heart dilatation, increase RVO and LVO, and improve LV and RV function, with no superiority of one modality over the other.

The echocardiographic results of this study indicated that SPAP decreased significantly in both groups at day 3 and 7 after ventilation compared to its value at day 1 with no significant difference between the two groups. In harmony with our results, Vijay Kumar et al. [14] included 40 neonates with PPHN and reported SPAP significantly decreased after treatment with either sildenafil or bonestan on the third and seventh day. This was expected to happen as ventilation with improved oxygenation aided by sildenafil helped to reduce PVR and consequently decreased SPAP. Interestingly, neonates on HFOV didn’t differ from those on CMV regarding SPAP decrement suggesting that HFOV has no negative impact on pulmonary pressure.

Our study revealed that TRPG decreased significantly in both groups at day 3 and 7 with no significant difference between the two groups. This decrease coincided with the decrease in SPAP. The tricuspid valve increases with the enlargement of the right atrium and ventricle, eventually delaying the opening of the tricuspid valve and closing it in advance and increasing the pressure and TR, hence decreasing SPAP with decreasing RV dilatation would definitely decrease TR.

Moreover, measurements of the right side of the heart in the form of RVD, RAD, and MPA significantly decreased at day 7 compared to their measurements at day 1 and day 3, with no significant difference between the two groups. The increased PVR in neonates with PPHN leads to an increase in right ventricular load, right ventricular, right atria, and pulmonary artery dilatation [15, 16]. Decreasing SPAP and TRPG from day 3 resulted in a gradual decrease of the measurements of the right side of the heart by day 7, as dimensions took more time to decrease compared to the decrease in pressure.

In our current study, RV systolic function in the form of TAPSE increased significantly at day 3 and 7, with no significant difference between the two groups. Increased PVR with consequent right ventricular overload and dilatation leads to involvement of right heart function, so that both ejection and filling time decline, coronary arterial blood perfusion is reduced, and myocardial cells are damaged, eventually resulting in a decline in the whole function of the right ventricle with more dilation of the right ventricle and atrium. A study by Papaioannou et al. [17] of ventilated patients with PH identified lower TAPSE in patients with declining LV function and/or increased RV filling pressure, indicating that either a reduction in contractility, or myocardial stretch from an acute increase in preload could also depress TAPSE.

Our study revealed that LVESD, LVEDD, LV FS, and LV EF significantly increased in both groups at day 7 compared to their values at day 1 and day 3, with no significant difference between the two groups. Increased PVR may lead to RV dilatation, causing leftward deviation of the interventricular septum, thereby reducing the LV cavity size and compliance with decreased LV preload, which explains that the right heart is dilated but the LV is not (due to reduced preload to LV and septal shift into the LV). This will further compromise left heart filling, and both systolic and diastolic function. This phenomenon is termed ‘interventricular interaction’ and has been demonstrated in animal models as well as adult patients of pulmonary hypertension. If the clinical state remains uncorrected, the LV ultimately decompensates causing critically low cardiac output and systemic hypoperfusion. When RV returned to its normal diameter that would correct septal shift into LV, increasing its diameter with subsequent increased preload, which resulted in improvement of LV function.

In our current study, SVC flow showed no significant changes with follow up with no significant difference between the two studied groups. SVC reflects venous return from the upper part of the body. PPHN and SVC flow were not yet directly associated in any published literature. SVC flow has been proposed as a surrogate measure of cerebral blood flow, and it has been associated with short-term and long-term outcomes in neonates as found by Stensballe et al. [18]. However, and Miletin et al. [19] have reported an association between low SVC flow in the first 24 h and IVH and/or neonatal death in neonates. No doubt, this generated a lot of interest in studying SVC flow and its hemodynamic impact in the neonatal period [20].

In our current study, RVO and LVO increased significantly in both groups, where RVO significantly increased at day 3 and 7 while LVO significantly increased at day 7 only, and this increase was more significant in the CMV group. Increased myocardial contractility, increased preload, or decreased PVR can all lead to an increase in RVO and LVO. Since the LV FS and LVEF improved significantly, increased myocardial contractility shares in this improvement. Moreover, preload to the left ventricle increased due to decreased SPAP, leading to an improvement of the venous return and raising the left ventricular preload and output. Aggarwal et al. [21] reported that LVO, RVO, and LV FS were significantly lower in neonates with PPHN compared to controls, suggesting that the reduced output was due to poor venous return.

Jain et al. [22] stated that, although different etiologies and underlying cellular mechanisms can lead to PPHN, its clinical phenotype often remains the same. The primary physiologic abnormality in PPHN is increased PVR, exposing the right ventricle to high afterload. Significant or prolonged exposure to increased afterload often leads to RV dysfunction. The coexistence of a high PVR and RV dysfunction may result in critically low pulmonary blood flow. Pulmonary hypoperfusion may cause significant ventilation-perfusion mismatch and a reduced venous return to the left atrium (left ventricle preload). In addition, patients often develop hypoxia and acidosis that can impede myocardial performance and further reduce pulmonary blood flow. Additionally, Sehgal et al. [23] found that systemic hypotension was seen very commonly in neonates with PH. Hypotension in this setting is likely to be multifactorial, right ventricular dysfunction with reduced pulmonary blood flow. Left ventricular dysfunction as part of disease severity or concurrent hypoxia ⁄ acidosis and reduced venous return because of the need for high ventilatory settings may be contributory factors. Leftward indentation of the interventricular septum might also contribute to low left ventricular capacity.

In our present study, HR significantly decreased in both groups, especially at days 3 and 7 compared to day 1, with no significant difference between the two groups. Going with our result, McNamara et al. [24] found that there was a significant decrease in HR after treatment of PH and reduction of pulmonary artery pressure. It is impossible to tease out whether this is a direct effect of sildenafil on the pulmonary vascular bed or secondary to the improvement in myocardial performance and oxygenation. LV usually compensates for reduced preload by increasing its contractility and HR; hence, after improvement of LV preload, HR decreased significantly.

SBP, DBP, and MBP in both groups significantly increased at day 7, with no significant difference between the two groups. The improvement of both systolic and diastolic blood pressure could be explained by reducing pulmonary afterload, improving RV compliance, and increasing myocardial contractility, hence increased cardiac output and blood pressure [24].

Regarding cranial ultrasound findings in our study, RI of the middle cerebral artery significantly decreased in the HFOV group only at day 7, reflecting an increase of cerebral blood flow with no significant change in the CMV group on follow-up.

Vasoconstriction at constant flow or increased flow can both cause an increase in cerebral blood flow velocity. It seems likely that the increase in cerebral blood flow velocity was caused by an increase in both SBP and LVO that was more pronounced in the CMV group. Changes in LVO may have a great impact on cerebral blood flow in neonates due to their immature cerebral autoregulation. There have been reports of an increased incidence of intraventricular hemorrhage due to HFOV, which could also be explained by an increase in cerebral perfusion pressure. Nevertheless, the incidence of IV hemorrhage in our study was comparable in both groups.

Our neonates with PPHN were managed with CMV and HFOV without targeted volume e.g. volume guarantee (VG). This is very important as VG ventilation may have a different impact on hemodynamics by altering venous return, pulmonary artery pressure, and ventricular function [25]. VG ventilation increases intrathoracic pressure, which may reduce venous return and right ventricular preload, potentially lowering cardiac output, particularly in preterm infants with limited myocardial reserve. In addition, excessive tidal volumes or elevated mean airway pressure may cause alveolar overdistension, leading to compression of pulmonary capillaries and increased pulmonary vascular resistance [25]. In infants with PH, further elevation of PVR may aggravate right ventricular afterload, impair right ventricular performance, and worsen right-to-left shunting. Consequently, careful optimization of tidal volume and positive end-expiratory pressure is essential to avoid hemodynamic compromise in this vulnerable population [26, 27].

Inotropic support represents another important determinant of neonatal hemodynamics. Inotropic support was provided according to the clinical needs of each neonate. Some infants did not require inotropes, whereas others received one or more agents, including dopamine, dobutamine, or milrinone. Dopamine may increase SVR and BP, enhancing coronary perfusion and myocardial performance. Dobutamine primarily augments contractility and SV, while milrinone reduces afterload and improves ventricular relaxation. These pharmacological effects may increase RVO and LVO independent of ventilation mode. However, in the present study, the proportion of neonates requiring inotropic support did not differ significantly between HFOV and CMV groups, suggesting balanced myocardial support and limiting their effects on the observed improvement in ventricular output.

These findings should be interpreted within the context of our local practice, where sildenafil is used as the first-line therapy for PPHN. Inhaled nitric oxide, widely considered the first-line treatment in high-resource settings, was not routinely available. Although both agents reduce PVR, their pharmacodynamics differ,. Therefore, the external generalizability of our results may be limited. Sildenafil could influence pulmonary vascular resistance, right ventricular output, and left ventricular preload, and therefore represents a potential confounding factor in interpreting the hemodynamic outcomes.

Limitations of the study: small sample size and short duration of follow-up. Hemodynamic measurements were not analyzed based on inotrope use, type, or dose. Given the small sample size and variability in the type and dosing of inotropes, subgroup analysis would have reduced statistical power and could yield unreliable results. Future studies with larger cohorts should adjust for pharmacologic cardiovascular support to better define the independent impact of ventilatory strategy on neonatal hemodynamics. The relatively large number of secondary endpoints was analyzed, which raises the possibility of type I error due to multiple comparisons.

## Conclusion

HFOV and CMV have positive comparable effects on hemodynamics in full-term neonates with PPH. However, LVO and RVO improved more significantly in neonates on CMV. Moreover, HFOV had a better impact in decreasing the RI of the middle cerebral artery than CMV in these neonates.

## Data Availability

Data are available from the corresponding author on reasonable requested.

## Abbreviations

CMV: Conventional mechanical ventilation
DBP: Diastolic blood pressure
FIO_2_: Fraction of inspired oxygen
HFOV: High frequency oscillatory ventilator
HR: Heart rate
IVH: Intraventricular hemorrhage
LVEDD: Left ventricular end-diastolic dimension
LV EF: Left ventricular ejection fraction
LVESD: Left ventricular end-systolic dimension
LV FS: Left ventricular fraction shortening
LVO: Left ventricular output
MAP: Mean airway pressure
MCA: Middle cerebral artery
MPA: Mean pulmonary artery
MBP: Mean blood pressure
PAAT: Pulmonary artery acceleration time
PEEP: Positive end-expiratory pressure
PIP: Positive inspiratory pressure
PPHN: Persistent pulmonary hypertension of neonates
PVR: Pulmonary vascular resistance
RAD: Right atrial diameter
RI: Resistance index
RVD: Right ventricular diameter
RVO: Right ventricular output
SBP: Systolic blood pressure
SD: Standard deviation
SPAP: Systolic pulmonary artery pressure
SVC: Superior vena cava
TAPSE: Tricuspid annular plane systolic excursion
TR PG: Tricuspid regurge pressure gradient
VG: Volume guarantee
VTI: Velocity time integral
